# Intracellular K^+^ and water content in human blood lymphocytes during transition from quiescence to proliferation

**DOI:** 10.1038/s41598-019-52571-1

**Published:** 2019-11-07

**Authors:** Irina Marakhova, Valentina Yurinskaya, Nikolay Aksenov, Valeriy Zenin, Alla Shatrova, Alexey Vereninov

**Affiliations:** Department of Intracellular Signaling and Transport and Laboratory of Cell Physiology, Institute of Cytology, Russian Academy of Sciences, St-Petersburg, Russia

**Keywords:** Cell growth, Permeation and transport

## Abstract

Many evidence shows that K^+^ ions are required for cell proliferation, however, changes in intracellular K^+^ concentration during transition of cells from quiescence to cycling are insufficiently studied. Here, we show using flame emission assay that a long-term increase in cell K^+^ content per g cell protein is a mandatory factor for transition of quiescent human peripheral blood lymphocytes (PBL) to proliferation induced by phytohemagglutinin, phorbol ester with ionomycin, and anti-CD3 antibodies with interleukin-2 (IL-2). The long-term increase in K^+^ content is associated with IL-2-dependent stage of PBL activation and accompanies the growth of small lymphocytes and their transformation into blasts. Inhibition of PBL proliferation with drugs specific for different steps of G0/G1/S transit prevented both blast-transformation and an increase in K^+^ content per cell protein. Determination of the water content in cells by measuring the density of cells in the Percoll gradient showed that, unlike the K^+^ content, the concentration of K^+^ in cell water remains unchanged, since water and K^+^ change in parallel. Correlation of proliferation with high cell K^+^ and water content has been confirmed by the data obtained in comparative study of PBL and permanently cycling Jurkat cells. Our data suggest that K^+^ is important for successful proliferation as the main intracellular ion that participates in regulation of cell water content during cell transition from quiescence to proliferation. We concluded that high K^+^ content in cells and the associated high water content is a characteristic feature of proliferating cells.

## Introduction

Intracellular monovalent ions such as K^+^, Na^+^, Cl^−^ are important for successful proliferation^1–7^. Inhibition of membrane-associated ion channels and pumps as well as ion co-transporters induces cell cycle arrest and stops proliferation^8–11^. An involvement of monovalent ions in regulation of cell proliferation is demonstrated by the proliferation-related changes in the profile of the transcribed mRNAs coding ion channels and transporters^12–17^. Monovalent ions may be involved in signaling network in cycling cell: changes in concentrations of cellular ions regulate pH_i_, membrane potential and the activity of some cell proteins with important function in the cell cycle progression^18–21^.

Our attention is focused on the role of intracellular K^+^ in proliferation. The asymmetric distribution of Na^+^ and K^+^ is a characteristic feature of most animal cells and higher cell K^+^ conсentration is necessary for the higher cell proliferation^22–24^. It was shown on Ehrlich ascites cells that K^+^ concentration in cell water remains unchanged during cell cycle^21^. Meanwhile, we found significant changes in K^+^ content (K_i_) in proliferating cultures of permanent cell lines: K_i_ calculated per g of cell protein content declined during culture growth from low to high density^7,25^. These changes in K_i_ were not accompanied by increasing cell Na^+^ content (Na_i_) and occurred simultaneously with a decrease in cell proliferation. We also found age-dependent decrease in K_i_ per g of cell protein in growing cultures of human mesenchymal stem cells^26^. Of interest, cell K^+^ content assayed by X-ray fluorescence spectrometry and mass spectrometry differs considerably between human pluripotent cells and non-pluripotent cells^27^.

Relationship between changes in K_i_ and proliferation was investigated in activated human peripheral blood lymphocytes (PBL)^28,29^. PBL represents an adequate model for investigating ion changes associated with cell transition from quiescence to proliferation. In PBL, stimulated by phytohemagglutinin, K^+^ content calculated per cell protein has been found to increase during the prereplicative stage^28^. In this study, we present new evidence that in human PBL, stimulated to exit from quiescence (G_0_) to cell cycle, K^+^ content per cell protein rises gradually in the course of IL-2-dependent transition of small resting T lymphocytes from G_0_ into large blasts and DNA synthesis. We also show that cell water content per cell protein calculated from cell buoyant density, is higher in activated than in resting PBL and cellular K^+^ concentration does not change during lymphocyte transition to proliferation. It is concluded that high cell K^+^ content per cell protein content as a sign of higher cellular hydration is a hallmark of cell proliferation and transformation.

## Results

### Cell K^+^ content in human PBL activated to transition from quiescence to proliferation

**Figure 1:**
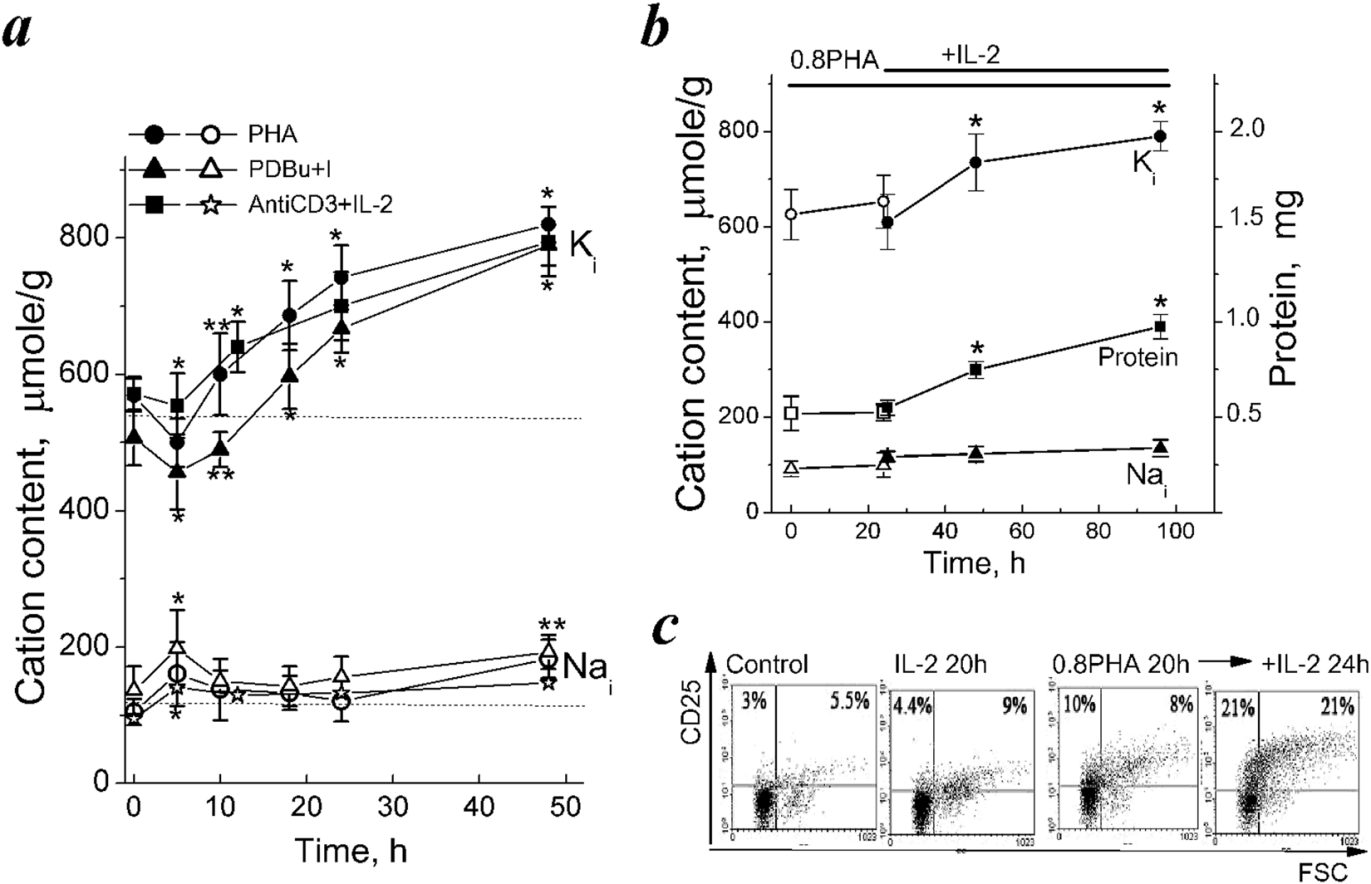
Cell K^+^ and Na^+^ contents in human PBL activated to transition from quiescence to proliferation. (**a**) Changes in cell K^+^ (K_i_) and Na^+^ (Na_i_) contents in isolated human PBL stimulated by PHA (10 µg/ml), or PDBu (10 nM) with ionomycin (I, 500 nM) or anti-CD3 (3.5 µg/mL) with IL-2 (100 U/mL). K_i_/g (dark symbols) and Na_i_/g (light symbols) were analyzed at definite time points by flame emission photometry. (**b**) IL-2 induces a long-term increase in K_i_ and cell protein content in competent PBL. Isolated PBL were incubated with non-mitogenic PHA (0.8 µg/mL) for 20 hours, then IL-2 (100 U/mL) was introduced into cell culture. Data are means ± SEM of nine (**a**, PHA and PHA + I), six (**a**, anti-CD3 with IL-2), or five (**b**) experiments performed triplicate. Significant difference from the initial value at *time 0* (resting PBL) was tested by one-way ANOVA with Tukey’s post hoc tests, *P < 0.01, ^**^P < 0.05. (**c**) Costimulation with non-mitogenic PHA and IL-2 is accompanied by increased CD25 expression. The representative data of one experiment from five. Control – non-stimulated, resting PBL.

First, we confirmed our previous report that in human PBL stimulated by mitogenic PHA transition from quiescence to proliferation is accompanied by long-term increase in cell K^+^ content^28^. Figure 1a shows that for the first 5 hours after addition of PHA (10 µg/mL) to resting PBL, there occurs a decrease in K_i_ that is followed by its increase up to 48 hour. An initial decrease in K_i_ takes place simultaneously with an increase in Na_i_. It has previously been established, that rapid reciprocal changes in Na_i_ and K_i_ as well as enhanced ion fluxes via Na pump and ion channels represent an early ion response as a part of initial T-cell receptor [TCR] activation by antigen^1,2,30^. As seen in Fig. 1a, at later stages of PBL activation, a significant increase in K_i_ was observed, whereas Na_i_ increased during the first 2–5 hours after stimulation and later it does not change or slightly increased to the end of second day. In our study, to evaluate changes in K_i_ and Na_i_, we normalized analytically measured cell ion content per cell protein content in the same culture. In cell biology studies, such evaluation is widely used for estimating intracellular contents of ions because of uncertainties in measuring cell volume and water content in proliferating cells. Indeed, in asynchronous growing cultures, cycling cells have different sizes and it is impossible to compare cells from different established lines as well as cells of different origin.

Here, we present new evidence that in activated PBL, an increase in K_i_ per cell protein content (K_i_/g) accompanies PBL transition from quiescence to proliferation. In resting T cells, calcium ionophores and phorbol esters are capable of triggering two early signalling cascades, such as intracellular Ca^2+^/NF-AT and protein kinase C/NF-kB, that are sufficient to induce Go/G1/S transit^31,32^. In our experiments, phorbol 12,13-dibutyrate (PDBu, 10 nM) and ionomycin (500 nM) were capable of inducing proliferation in isolated human PBL (Table 1). As with mitogenic PHA, in the presence of PDBu and ionomycin an early decrease of K_i_ was followed by a sustained elevation to 760 ± 30 μmole/g at 48 hour (Fig. 1a, Table 1). After early increase during the first 5 hours, Na_i_ remains stable and began to increase to 48 hour only (Fig. 1a).

**Table 1:** Cell K^+^, Na^+^ and water contents, and K^+^ concentration in human PBL during transit from quiescence to proliferation.

	Incubation	Cation content, µmole/g		Cell water, mL/g protein	[K_i_], mM	S + G2 + M, %
		K_i_	Na_i_			
1	Resting PBL	613 ± 12	110 ± 13	4.83 ± 0,15	126	0,7 ± 0.01
2	PHA, 5 h	500 ± 24	215 ± 23	ND	ND	ND
3	PHA, 24 h	742 ± 21	140 ± 12	6.31 ± 0,37	118	1.9 ± 0.2
4	PHA 48 h	820 ± 47	173 ± 21	7.49 ± 0.41	110	38.9 ± 4.5
5	PDBu + I	760 ± 30	193 ± 25	ND	ND	41.5 ± 5.8
	Anti-CD3 + IL-2, 24 h	700 ± 50	133 ± 48	6.1 ± 0.25	115	1.3 ± 0.02
6	Anti-CD3 + IL-2, 48 h	785 ± 51	148 ± 16	7.03 ± 0.6	114	40.8 ± 3,1
7	Competent PBL (0.8PHA, 24 h)	648 ± 37	103 ± 16	ND	ND	1.3 ± 0.02
8	Competent PBL + IL-2, 48 h	790 ± 31	135 ± 17	ND	ND	31.0 ± 5.4
9	PHA + CsA, 48 h	548 ± 41	184 ± 8	ND	ND	18.1 ± 2.1
10	PHA + WHI-P131, 48 h	516 ± 29	177 ± 19	ND	ND	11.0 ± 3.9
11	Competent PBL + (IL-2 + WHI-P131) 24 h	599 ± 42	169 ± 20	ND	ND	5.4 ± 1.7
12	Jurkat T cells	804 ± 101	141 ± 32	5,84 ± 0.35	138	37,4 ± 3,2

From: Intracellular K^+^ and water content in human blood lymphocytes during transition from quiescence to proliferation.

Isolated PBL were incubated with 10 μg/ml PHA, or with phorbol ester (PDBu,10 nM) and ionomycin (I, 500 nM), or with anti-CD3 antibodies (3.5 µg/mL) and IL-2 (200 U/mL) for 24 or 48 h. Data for K_i_, Na_i_, cell protein and DNA cytometry are means ± SEM (p ≤ 0.05) of nine (PHA and PDBu + I), four (PHA with inhibitors), six (anti-CD3 antibodies with IL-2), five (competent PBL with IL-2 or with WHI-P131) experiments with PBL and of five experiments with Jurkat T cells. In each experiment, all the values were determined from 3 cultures of PBLs from one donor. Data for cell water per g protein, *v*_prot_, was calculated as *v*_prot_ = (1 − ρ/ρ_dry_)/[0.72(ρ − 1)], taking the density of cell dry mass as 1,38 g/mL and the ratio of protein to dry mass as 72%. Means ± SEM of three independent experiments on PBL from three donors.

To initiate prolνiferation in resting PBL we also used antibodies for CD3 (anti-CD3) together with interleukin-2 (IL-2). This combination is widely used to distinguish signalling pathways which are responsible for early T cell activation and cell cycle progression^32^. As seen in Fig. 1a, in the presence of anti-CD3 (3.5 µg/mL) and IL-2 (100 U/mL), the time course of K_i_/g was similar to that observed with mitogenic PHA or PDBu and ionomycin: the initial decrease in K_i_/g (that was smaller than with other stimuli) was followed by gradually increasing K_i_/g during the next day without significant changes in Na_i_/g. Altogether, above data indicate that as compared to resting cells, in late activated PBL K_i_/g is higher depending on stimuli as well as on donor. In comparison with resting cells, in activated PBL, K_i_ per g cell protein ratio was 25–30% higher.

### Long-term increase in intracellular K^+^ content is associated with IL-2-dependent cell cycle progression and growth of quiescent T cells into blasts

To identify a stage of G_0_/G_1_/S transition when K_i_ is increased, we used two-step activation of quiescent PBL^33,34^. There is a possibility to differentiate between the initial T cell activation induced by signalling from TCR and the cell cycle progression when formation of the high affinity IL-2 receptor (IL-2R) takes place^35,36^. In two-step activation experiments we used submitogenic concentrations of PHA (0.8 μg/ml, 20 hours) to make PBL responsive to exogenous IL-2, and determined CD25 expression as a marker for IL-2Rα-chain and IL-2-dependent progression of stimulated T cells.

In PBL obtained from different healthy individuals PE-labeled CD25 markers could be detectable in 0.5–5% of total PBL pool. In the presence of submitogenic PHA the number of CD25^+^ cells began to increase as early as in 5 hour, however, in 24 h the percentage of CD25^+^ cells rose to 18.6 ± 1.7% only (Fig. 1c). In such PHA-treated PBL, the exogenous IL-2 (100 U/ml) increased the number of CD25^+^ cells to 40.3 ± 2.5 at 24 hour and induced cell cycle progression (Fig. 1c, Table 1).

In PHA-treated, competent PBL, K_i_ did not exceed 650 ± 55 μmole/g (Table). Addition of IL-2 to competent PBL induced the sustained elevation of K_i_ to 790 ± 31 μmole/g during the next 48 hour (Fig. 1b, Table 1). In resting PBL, IL-2 alone did not induce cell proliferation, CD25 expression as well as any changes in K_i_ (Fig. 1c). These experiments showed that in activated PBL, the long-term increase in K_i_/g is associated with IL-2-dependent cell cycle progression.

**Figure 2:**
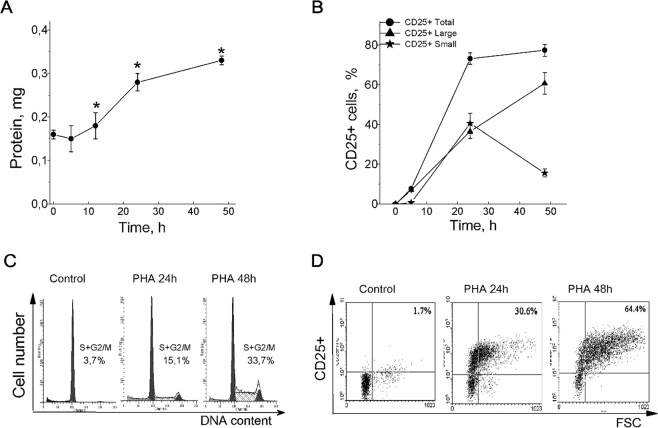
Long-term increase in cell K^+^ content precedes DNA synthesis and accompanies transformation of small T cells into large blasts. (**A**) The protein content increase in PBL stimulated by PHA (10 µg/ml) for 48 hours. Data are means ± SEM of twelve experiments performed triplicate. *Significant difference from the initial value at *time 0* (resting PBL) was tested by one-way ANOVA with Tukey’s post hoc tests, *P* < 0.05. (**B**) The time course of CD25 expression on small and large PBL stimulated by PHA. Data are means ± SEM of one representative experiment of three. (**C**) Flow cytometric analysis of cell cycle phase distribution in PHA-stimulated PBL: the percentage of cells in S and (G_2_ + M) phases. (**D**) The time course of CD25 expression in PBL stimulated by PHA for 48 hour. Resting or PHA-stimulated PBL were stained with PE-labeled CD25 Abs and analyzed by flow cytometry. (**C**) and (**D**) – representative data of three experiments with PBL from different donors. Control - non-stimulated, resting PBL.

To elucidate the relationship between K_i_ changes in activated PBL and cell proliferation, we compared the time-dependent changes in K_i_ with some parameters indicating the G_0_ exit and cell cycle progression. Flow cytometry analysis showed that in PHA-stimulated PBL culture, there were no more than 10–15% of cells in (S + G2/M) phases at 24 hour and a maximum of cells (up to 45%) in (S + G2/M) phases was at 48 hour or later depending on donor (Fig. 2C). In stimulated PBL, for two days, total cellular protein increased 2–2.5 times, with the greatest increase in cell protein mass between 12 and 30 hours (Fig. 2A). To assess cell growth we also analyzed FSC/FITC cytograms that were informative not only for dynamic of CD25 surface expression but also allow estimating the number of large CD25^+^ cells as compared to small cells in lymphocyte population. As revealed by FACS analysis, the increased CD25 expression was detectable at 5 hour after PHA addition (Fig. 2D). Later, the percentage of CD25^+^ cells was substantially increased from (8.7 ± 0.8) after 5 hour to (36.6 ± 3.5%) after 24 hour, and to the end of the second day, T cell population becomes represented mainly by large CD25^+^ cells (Fig. 2B). When comparing the cultures stimulated by PHA, PDBu with ionomycin or anti-CD3 with IL-2, we conclude that the elevation in K_i_ per g cell protein precedes DNA synthesis and accompanies transformation of normal small T cells into large blasts.

### Intracellular K^+^ content and inhibition of proliferation in activated human PBL

To highlight further the relationship between intracellular K^+^ and cell proliferation we tested some drugs specific for different steps of G0/G1/S transition in activated T cells. First, we used cyclosporine A (CsA) to prevent IL-2 production in mitogen-stimulated PBL. Immunosuppressive action of CsA is due to inactivation of calciuneurin, Ca^2+^-dependent phosphatase, that activates nuclear factor of activated T cells (NFAT) required for expression of IL-2 gene^37^. CsA has been shown to prevent induction of T cell proliferation.

**Figure 3:**
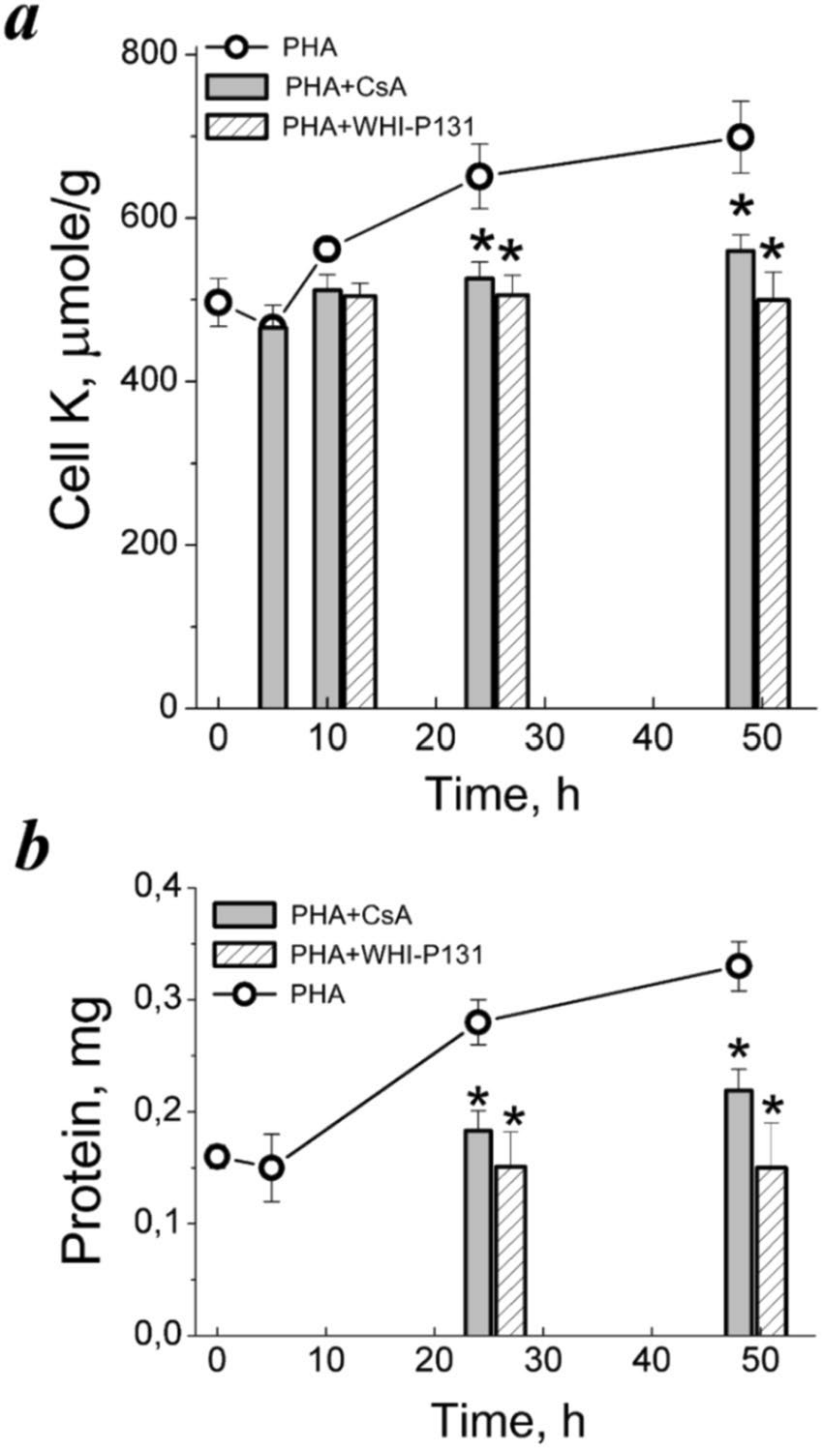
Cell K^+^ content and inhibition of proliferation in PHA-stimulated PBL. (**a**) Time-course of K_i_/g in PBL stimulated with PHA alone or in the presence of drugs specific for initial (CsA) or late (WHI-P131) stages of PBL activation. (**b**) Anti-proliferative doses of CsA and WHI-P131 inhibit growth of PHA-stimulated PBL. Cells were cultivated with 10 µg/mL PHA without or with 80 µM WHI-P131 or 1.0 µg/mL CsA for 48 and at definite time of points were analyzed for K_i_ by flame emission photometry and for protein content by Lowery procedure. Data are means ± SEM of one representative experiment performed triplicate. *Significant difference relative to PBL stimulated by PHA without drugs during the same time (*t*-test, *P* < 0.05).

CsA (1 µg/mL) was added to PBL culture 1 hour prior to PHA. Cell cycle analysis revealed a decrease in S phase in CsA-treated PHA-stimulated PBL (Table 1). CsA inhibited also an increase in protein content and a long-term elevation of K_i_ in activated cells but does not affect the initial decrease of K_i_ in response to PHA (Fig. 3a,b).

Next, we tested drugs specific for IL-2R signaling in T cells. We used WHI-P131 as an inhibitor of IL-2R-associated JAK3/STAT5 signaling and IL-2-dependent T cell proliferation^38^. In recent study, we established that 80 μM WHI-P131 did not affect survival of resting PBL^39^. In PHA-stimulated PBL, WHI-P131 suppressed cell growth and DNA synthesis induction (Fig. 3b, Table). WHI-P131 did not affect K_i_/g in resting as well as in competent PBL (data not shown). After 48 hour of PHA stimulation in the presence of 80 μM WHI-P131, K_i_ was 516 ± 29 μmole/g instead of 820 ± 47 μmole/g in cells activated without WHI-P131 (Fig. 3a, Table 1). These experiments again showed the close relationship between cell growth and cell cycle progression on the one hand and K_i_ per g cell protein ratio as a characteristic of ionic homeostasis of proliferating cell on the other hand.

### Buoyant density of resting PBL compared with activated PBL and leukemia T cell Jurkat

In view of the significant increase in K_i_ during transition from quiescence to proliferation it was important to know whether K^+^ concentration (i.e., cell K^+^ content per cell water content) changes in activated PBL. Therefore, we investigated whether cell water content was changed in quiescent PBL stimulated to enter cell cycle. Cell water content was determined by measurements of the buoyant density of cells in discontinuous Percoll density gradients. This is the most sensitive and reliable of all currently existing methods for determination of water content in native cell^40^.

**Figure 4:**
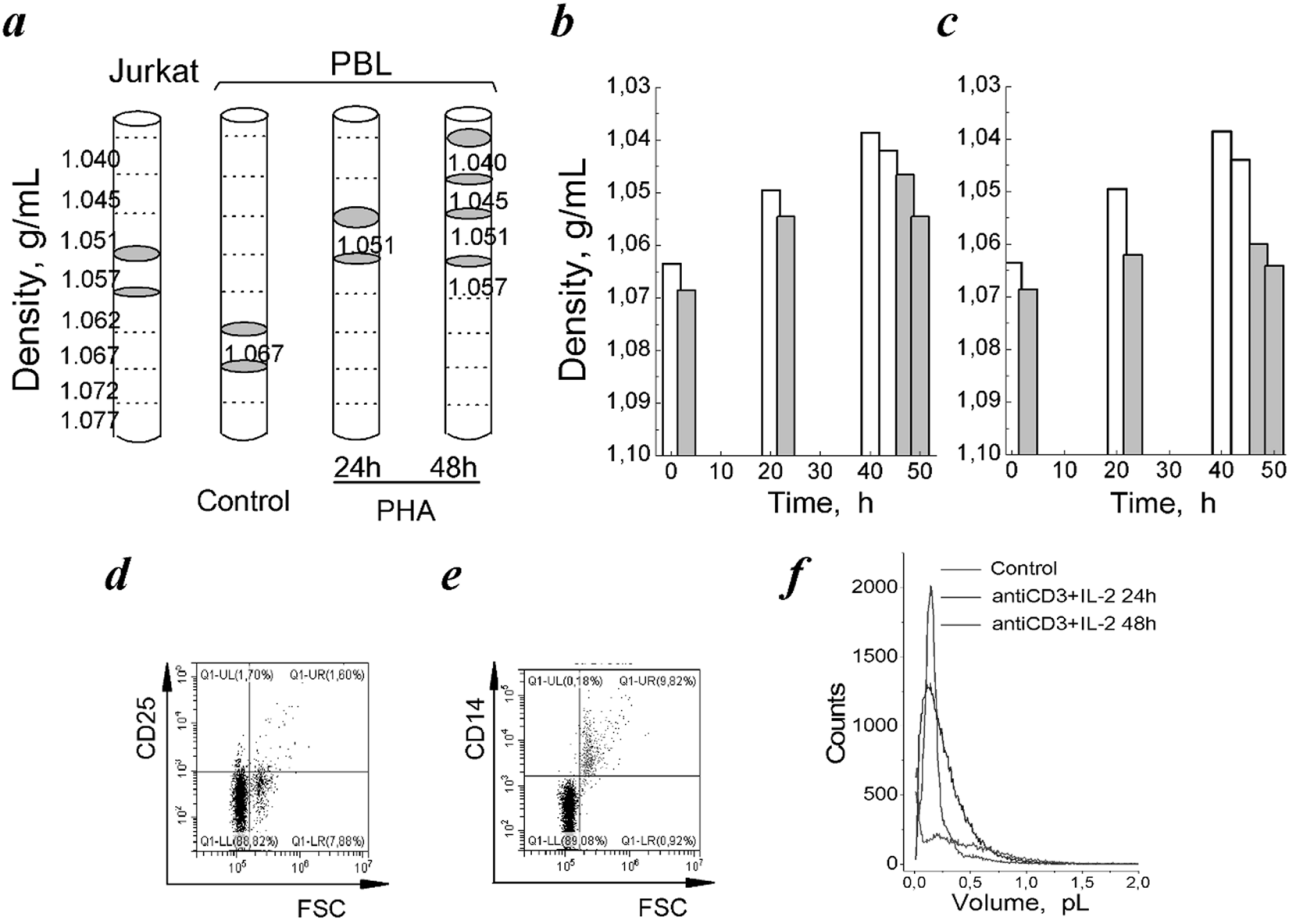
Buoyant density of resting PBL compared to activated PBL and leukemia T cell Jurkat. (**a**) Schematic illustration of the cell distribution in discontinuous Percoll gradient. Grey areas indicate the location of T cells Jurkat, resting and PHA-activated PBL isolated for analysis. (**b**,**c**) Changes in buoyant density in PBL stimulated by PHA (**b**) and anti-CD3 (3.5 µg/mL) with IL-2 (100 U/mL) (**c**) for 24 and 48 hours, each column corresponds to a separate layer. One representative experiment of three. (**d**,**e**) Flow cytograms of resting PBL in coordinates CD25/FSC (**d**) and CD14/FSC (**e**). The representative cytograms of seven experiments on PBL from different donors. (**f**) The representative cell volume distributions by Scepter Counter in PBL, resting and stimulated by (anti-CD3 and IL-2) mixture for 24 and 48 hours. Control - non-stimulated, resting PBL.

Isolated human PBL stimulated by PHA or by anti-CD3 with IL-2 were used for experiments. Figure 4a demonstrates schematically the results of separation of resting and activated PBL in Percoll gradient in one representative experiment of three. The resting PBL were distributed mainly in the high density region occupying two layers - 1.062–1.067 and 1.067–1.072 g/mL. Stimulation of PBL by PHA led to shifting cell populations towards lower densities and at 48 hour two low-density (1.039 and 1.042 g/mL) and two high-density (1.0465 and 1.0545 g/mL) populations were isolated (Fig. 4b). Similar changes in buoyant density were found in PBL stimulated by anti-CD3 with IL-2. As with PHA, after 24 hour stimulation the buoyant density was decreased to 1.0495 and 1.0620 g/mL, at 48 hour the distribution area of activated PBL in density gradient becomes wider and two low-density and two high-density populations appeared (Fig. 4c).

The presence of populations with different buoyant densities in resting PBL is more likely due to the heterogeneity of lymphocytes that are isolated from the whole fresh blood. According to the Basic Separation Protocol for human peripheral blood, after gradient separation procedure up to 40% of the mononuclear cells are monocytes^41,42^. In early studies of leukocytes separation in Ficoll-Hypaque density gradient it was reported that the mean density of monocytes was higher that of lymphocytes^43^. Monocytes can be depleted from the mononuclear cell suspension, since they adhere to plastic surfaces and lymphocytes do not. Usually, after depletion of monocytes by plastic adherence, 85–95% of the mononuclear cells are lymphocytes.

We tried to distinguish between lymphocytes and monocytes in view of differences in their sizes. Monocytes may be defined by flow cytometry on the basis of light scatter properties. In our flow cytometry analysis, FSC/SSC plots demonstrated two populations in isolated PBL, the smaller of which (5–11%) might be represented by monocytes. From Fig. 4d, CD25-negative small cells in the left quadrant of cytogram are lymphocytes, whereas cells in right quadrant might be monocytes. These cells are CD14-positive as determined with FITC-anti-CD14 as a marker of monocytes (Fig. 4e). Based on these estimations monocyte subset in our experiment do not exceed 9–13%.

The increased heterogeneity of PBL stimulated to exit from quiescence is most likely associated with asynchronous cell growth. Isolated PBL consists of T cells (75–90%), B cells (5–15%), NK cells (5–10%). All these cells have many subsets based on their maturation, activation, expansion stages and are characterized by their own growth response to activating stimuli. For instance, NK cells are large granular lymphocytes of low cell density^44^. It is known, NK cells do not express cell-surface CD3.

The heterogeneity of activated PBL is clearly seen in the volume distribution histograms obtained with Scepter Cell Counter. As seen in Fig. 4f, in activated PBL the volume distributions expand both at 24 and 48 hours and the average cell volume are increased from 0.17 pL in resting cells to 0.39 and 0.41 pL after 24 and 48 hours of mitogen stimulation. FACS analysis also demonstrates that activated PBL are heterogeneous in their size (Fig. 2D). To the second day of stimulation, the lymphocyte population consists of small and large CD25^+^ cells and to the third day only T cell population becomes represented mainly by large cells (Fig. 2B).

Based on the buoyant density estimations the water content per g protein was calculated for resting and activated PBL. In resting PBL, the water content was found to be 5.16 ± 0.19 mL/g (n = 3) in light subpopulation and 4.61 ± 0,07 mL/g (n = 3) in heavy subpopulation. After 24 hour of cell stimulation by PHA the water content increased to 6.48 ± 0.56 mL/g (n = 3) in the lighter cells and to 5.51 ± 0.50 mL/g (n = 3) in the heavier cells, and after 48 hour of stimulation two subpopulations with water contents up to 8.55 ± 0.26 mL/g (n = 3) and 6.55 ± 0.30 mL/g (n = 3) were present. The higher water content was also found in PBL, activated by anti-CD3 with IL-2 (Table 1). The most significant increase in average water content (about 27%) was observed for the first activation day. Altogether, these data indicate that water content in activated PBL which exit from quiescent state to cell cycle is higher than in non-stimulated resting PBL.

In parallel, the buoyant density of human leukemic T cells Jurkat was estimated. It was found that taken from proliferating cultures (with S phase of 35–37%) these cells were mainly distributed in region of 1.057 and 1.051 g/mL (Fig. 4a). The calculated average water content was found to be 5.84 ± 0.35 mL/g (Table 1). From this, in transformed human T cells water content/g protein was higher than in quiescent human blood lymphocytes.

**Figure 5:**
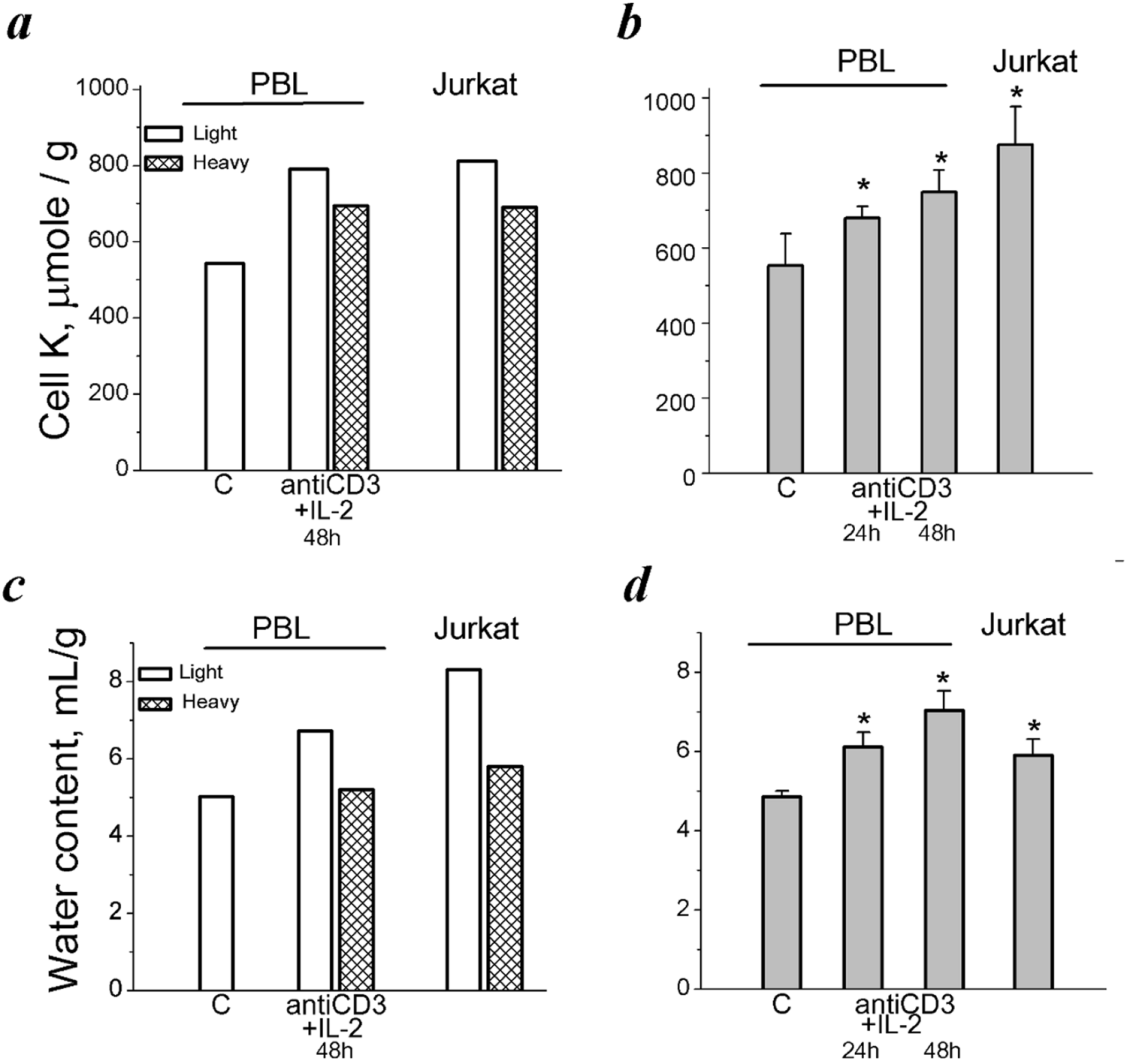
Cell K^+^ and water content in PBL with different buoyant densities as compared to unfractionated activated PBL and cycling T cells Jurkat. (**a**) K_i_/g in light and heavy cell populations. One representative experiment of four. (**b**) Changes in K_i_/g in total unfractionated cell populations. Data are means ± SEM of one representative experiment performed triplicate. *Significant difference relative to control (C) (*t-*test, *P* ˂ 0.05). (**c**) Water content in light and heavy cell populations stimulated with anti-CD3 with IL-2 for 48 hours and in proliferating T cells Jurkat. The same experiment as in (**a**). (**d**) Water content in human PBL stimulated from quiescence to proliferation and in proliferating T cells Jurkat. The same experiments as in (**b**). Data are means ± SEM of one representative experiment performed triplicate. *Significant difference relative to control (C) (*t-* test, P < 0.05).

Next, we determined K_i_ in populations of PBL with different buoyant densities. As found by flame emission measurements, the lighter subpopulations of activated PBL differed by K_i_ from heavy resting PBL. In representative experiment shown in Fig. 5a, in resting cells K_i_ was 542 µmole/g, whereas in activated PBL it was 791 µmole/g the (low-density population) and 699 µmole/g (the high-density population). In continuously proliferating leukemic T cells Jurkat K_i_ was found to be 818 and 691 µmole/g protein in the low- and high-density populations, correspondently (Fig. 5a). Resting and light and heavy subpopulations of activated PBL were also different in water content (Fig. 5c). These data are in good agreement with those which were obtained in parallel experiments on the total populations of isolated PBL: stimulation of resting PBL by anti-CD3 with IL-2 was accompanied by increasing both K_i_ per g cell protein and cell water content (Fig. 5b,d).

## Discussion

In this study we present evidence that cell transition from quiescence to proliferation is accompanied by gradual increase in intracellular K^+^ content per cell protein content and discuss the functional role of increasing cell K^+^ content in starting cell proliferation. In PBL stimulated by PHA, phorbol ester with ionomycin, or anti-CD3 antibodies with IL-2, long-term increase in K_i_ is associated with IL-2-dependent cell cycle progression when small resting T cells are transformed into large blasts. The close relationship between increasing K_i_/g and cell proliferation is confirmed in experiments with drugs which are specific for different steps of G0/G1/S transit and which in activated PBL prevents both the blastransformation and the long-term increase in K_i_/g.

Proliferation-related changes in cell K^+^ content, but not in cell Na^+^ content, were earlier found in growing cultures of permanent cell lines: under optimal culture condition, K_i_ per g cell protein gradually decreased during growth of culture to high density^7,25^. Recently, we have revealed that a decrease in K_i_ per g cell protein accompanies growth of human mesenchymal stem cells in culture^26^. The decrease in K_i_ per g cell protein is associated with the accumulation of cells in G1 phase of cell cycle and with the decrease in proliferation rate of cell culture.

Since there is an essential difference in K_i_ per g cell protein in quiescent and proliferating cells, the question arises whether intracellular K^+^ concentration is also changed and what can be the functional significance of increasing K_i_ during transition from quiescence to proliferation. We determined the cell water content per g cell protein by measuring the buoyant density of cells in the Percoll gradient and cell volume using a Coulter counter in resting and proliferating cells and found that a change in K_i_ per g cell protein is not followed by changing of K^+^ concentration in the cell water. We conclude that there are no significant differences in K^+^ concentration between quiescent and activated PBL.

It is known from the theory of monovalent ion distribution between animal cells and the medium that the amount of K^+^ in cell essentially depends on the amount of so called “impermeant (through cell membrane) anions” sequestered in cell. It is the amount of these anions in combination with Na, K ATPase pump that determines the water balance of the cell and the accumulation of K^+^ in the cells^45–55^. We can suggest that in activated PBL, an increase in dry mass (total cell protein) during blasttransformation is accompanied by an increase in the amount of impermeant anions per g dry mass, inevitably leading to an increase water influx to restore osmotic balance of cell with medium. Here, K^+^ as the major cellular osmolyte enters the cell so that an increase in K_i_ per g cell protein correlates always with an increase in cell water content per g cell protein, and therefore, the higher ratio of K_i_ per g cell protein indicate the higher cell water content/g protein ratio. Thus, in growing animal cell, increased cell K^+^ content per g cell protein indicates increased cellular hydration. This means, that despite the lack of significant concentration differences, K^+^ may be important for initiating cell proliferation as the intracellular ion that participates in regulation of volume and water content. Indeed, iincreasing evidence indicate that K^+^ channels of plasma membrane are involved in regulation of cell proliferation: their expression and function can be regulated by the cell cycle, and inhibition of K^+^ channel activity results in cell cycle arrest^12,26,27,56,57^.

To date, few studies are available on changes in K_i_ and water content in cells induced to proliferation. It was shown that in Ehrlich ascites (ELA) cells synchronized in G0/G1 by serum starvation and then released in serum-containing medium, increase in Na_i_ and Cl_i_ (not of K_i_) as well as in water content was observed in late G1/S transition^21^. Because of the concomitant change in water content, cell Na^+^ and Cl^−^ concentrations did not differ between G1 and S. It was concluded that Na^+^ regulates proliferation by controlling intracellular pH, while Cl^−^ regulates membrane potential and participate in cell volume regulation during S-phase.

When compared our data on water and K_i_ in quiescent PBL stimulated to proliferation with synchronized ELA cells we note some similarities in changes of cell water content. In both studies, water content/protein content ratio increases during cell activation, however, in released ELA cells, the increase in water content was observed in the late cell cycle transit, and it was smaller (about 11–12%) than in activated PBL (up to 45–49%). Differences in absolute values of water content changes between ELA cells and PBL are most likely explained by different functional status of cells. Namely, serum deprivation for 72 hours shifted ELA cells into nonproliferative state in which about 7% and 4% of cell population still remained in S and in G2/M phases, correspondently^21^. Upon reintroduction to standard culture conditions, ELA cells restored proliferation with maximal S phase (about 36%) as soon as in 16 hours.

Human peripheral blood lymphocytes are quiescent (G0) cells. Cellular quiescence is actively maintained: certain transcription factors acts as regulators of gene expression patterns that enforce the quiescent phenotype^58–60^. In quiescent PBL, antigen recognition with appropriate co-stimulation triggers exit from G0 state and further transit through cell cycle (G0 → G1/S/G2/M). In contrast to ELA cells released after starvation, antigen-activated PBL have long growth phase (48–72 hours) that precedes DNA replication. This time is required to increase cell size and metabolism. In activated PBL, prior S phase entry, total protein content was increased 2–2.5 times during 2 days whereas in ELA cells protein content did not differ between different samples or increased about 1.2 times 16 hours after release^21^. Significant differences in growth intensity correlates with differences in absolute changes in water content in PBL stimulated to proliferation from quiescence and in ELA cells released after starvation.

As to K_i_, our data on stimulated PBL contrasts with data on ELA cells^21^. As shown, in synchronized ELA cells, neither the concentration nor the content of K^+^ were changed during cell cycle progression. In contrast, in quiescent PBL stimulated to enter cell cycle, K_i_/g was significantly (by 20–30%) increased concomitant with an increase in cell protein content. As far as in activated PBL the water content was also increased, the intracellular concentration of K^+^ was not changed during transit from quiescence to proliferation. The main finding with activated PBL is that during the exit from quiescent state, an increase in cell K^+^ content occurs simultaneously with an increase in cell water content, thus resulting to higher cellular hydration.

Intracellular water is responsible for the conformations and functions of all biomolecules through direct interaction with their hydration shells and hydration changes affect cell functions at multiple levels including cell metabolism and gene expression. There is also considerable evidence that water of cancer cells is similar to that of embryonic tissue and consistently higher than that of normal cells of similar origin^61^.

In summary. In this study we describe proliferation-associated increase in cell K^+^ content per cell protein content concomitant with cell water increase during human lymphocytes transition from quiescence to proliferation and conclude that intracellular K^+^ concentration remains unchanged during G0/G1/S progression. Our data suggest that the functional meaning of long-term increase in cell K^+^ content during the transition from cell quiescence to proliferation may be that K^+^ as a dominant intracellular osmolyte is involved in the regulation of cell water content during cell growth and transformation and high cell K^+^ content per cell protein content (as a sign of higher cellular hydration) is a hallmark of cell proliferation and transformation. This conclusion seems to be of importance for all cells which are quiescent under homeostasis (lymphocytes, fibroblasts, stem cells) and respond to stimuli by exiting from quiescence and entering cell cycle.

## Methods

### Cells and experimental protocol

The research and all procedures involved human blood lymphocytes were performed in accordance with standards of the Declaration of Helsinki (1989) and approved by the Ethics Committee of the Institute of Cytology (No. 223/934 on the 27/10/2017). Lymphocytes were isolated from fresh venous blood of healthy adult donors. Written informed consent was obtained from all of the participants according to agreement between the Institute of Cytology RAS and the State Institution “Mariinsky Hospital” (permission number 2025/14, Saint-Petersburg).

For experiments, PBL were obtained by density gradient centrifugation over Histopaque (Histopaque-1077, Sigma), as described previously^28^. After depletion of adherent cells on plastic bottles, isolated lymphocyte population was >85% CD3^+^ cells. Isolated PBL were incubated overnight in RPMI-1640 medium with heat-inactivated AB IV Rh (+) human serum (5%) in CO_2_-air (5–95%) atmosphere at 37 °C without antibiotics. The next day the cell suspension (1.5 × 10^6^ cells/mL) was placed into vials, and stimulated by 10 μg/mL phytohemagglutinin M (PHA-M, Sigma, USA), or by 10 nM phorbol 12,13-dibutyrate (PDBu, Sigma, USA) and 500 nM ionomycin (I, Sigma, USA), or by 3.5 µg/mL anti-CD3 antibodies with human recombined IL-2 (100 U/mL, Biotex, Russia), or left unstimulated. In the «two-pulse activation experiments», PBL were treated with submitogenic concentrations of PHA (0.8–1.0 μg/mL) for 20 hours to induce “competence” and then the competent cells were stimulated by IL-2 (100–200 U/mL) to induce progression to the S phase. Lymphocytes of one donor (up to 400 × 10^6^ cells) were used in each experiment.

To compare the ion homeostasis and water content of quiescent and activated T cells with those of transformed T cells we use human leukemia T cell Jurkat (Russian Cell Culture Collection of the Institute of Cytology RAS, Saint-Petersburg). Jurkat cells were maintained in RPMI-1650 with 10% fetal calf serum (Biolot, Russia) containing 2 mM L-glutamine in CO_2_-air (5–95%) atmosphere at 37 °C without antibiotics.

### Intracellular cation measurements

Cellular K^+^ and Na^+^ content was measured by emission photometry in an air–propane flame using a Perkin-Elmer AA 306 spectrophotometer, as described previously^28^. In summary, the cells were pelleted in RPMI medium, washed five times with MgCl_2_ solution (96 mM) and treated with 5% trichloroacetic acid (TCA). TCA extracts were analyzed for ion content. TCA precipitates were dissolved in 0.1 N NaOH and analyzed for protein by Lowry procedure. The cell ion content was expressed as amount of ions per amount of protein in each sample analyzed.

### Analysis of cell water content

Cell water was determined by measurements of the buoyant density of cells in discontinuous Percoll gradients. The density gradient centrifugation method is a widely accepted method of measuring the average density of a cell population. In our experience, this is the most sensitive and reliable of all currently existing methods for determination of cell water. An isotonic Percoll solution was made by mixing Percoll (25 mOsm/kg, 1.130 g/ml; Pharmacia, Sweden) and PBS at a 9:1 volume ratio. Densities of 1.040–1.077 g/mL were prepared by diluting the isotonic Percoll solution in PBS at concentrations of 25–53% and tested by the refractive index measured using a Atago PR-RI Palette digital refractometer (Japan).

An eight-layered Percoll gradient was obtained by layering 0.25 mL of each Percoll solution in 95 mm-length tubes. The density step was about 0.005 g/mL. The concentrated cell suspensions (100 µL of 1–2 × 10^6^ cells) were placed on the top of the gradient and centrifuged for 20 min at 400 *g* (Eppendorf Centrifuge 5804, Germany). After centrifugation, the fractions were collected, placed into 1.5 mL Eppendorf tubes, suspended in the RPMI medium and used for determination of ion content and cell protein.

The water content per g protein, *v*_prot_, was calculated as *v*_prot_ = (1 − *ρ/ρ*_dry_)/[0.72(*ρ* − 1)], where *ρ* is the measured buoyant density of the cells and *ρ*_dry_ is the density of the cell dry mass, the latter taken as 1.38 g/ml. The ratio of protein to dry mass was taken as 0.72. The buoyant density of cells is a more sensitive and reliable measure for cell water content than all known techniques including methods using intra- and extracellular water markers.

### Cell size assay

Cell size was measured by Scepter cell counter equipped with a 40-μm sensor and software version 2.1, 2011 (Merck Millipore, Germany)^40^. Resting or stimulated with mitogens PBL were centrifuged at 2000*g* and re-suspended in Hank’s balanced salt solution. Cells were diluted to concentrations of (0.5–1) × 10^6^ cells/mL, which lie within the operating range of the instrument.

### Cell cycle analysis

FACS analysis was used to assess the proliferation of stimulated PBL and cultured T cell Jurkat^39^. Briefly, at definite time after mitogenic stimulation, PBL (10^6^ cells/mL) were suspended in PBS containing 200 µg/mL of saponin (Fluka, NY, USA) for 30 min, then washed and treated with 250 μg/ml ribonuclease (Sigma-Aldridge, MO, USA) and 50 μg/mL propidium iodide (Sigma, USA) in PBS for 30 min at 37 °C. Cell cycle analysis was performed with Coulter Epics XL Flow Cytometer (Beckman Coulter, CA, USA) and WinList and ModFit LT software (Verity Software House, USA). At least 10,000 cells were measured per sample.

### High-affinity IL-2R assay

To assess the expression of IL-2R, phycoerythrin (PE)-coupled CD25 antibodies as cell surface markers of the α-chain, and FACS analysis were used as described previously^39^. PBL were pelleted by centrifugation, rinsed once and suspended in PBS (10^6^ cells/mL) and stained with PE-labeled CD25 Abs (Invitrogen, USA). Mouse IgG-PE isotype control was used for assessing the background staining of cells. The percentage of CD25^+^ cells was determined after gating on lymphocytes. Cells were analyzed on a Coulter Epics XL Flow Cytometer (Beckman Coulter, CA, USA). Statistical analysis of results was performed according to the standard protocol of the data treatment Epics XL.

### Statistical analysis

All data are presented as the mean and standard error of the mean (SEM) from at least three independent experiments. Statistical significance was assessed using two-tailed Student’s *t*-test and one-way ANOVA with Tukey’s post hoc tests for multiple comparisons, *P* < 0.01 and *P* < 0.05. The specific details of each experiment are provided in the corresponding figure legends.
